# Mental Health in the Transit Context: Evidence from 10 Countries

**DOI:** 10.3390/ijerph19063476

**Published:** 2022-03-15

**Authors:** Maria Caterina Gargano, Dean Ajduković, Maša Vukčević Marković

**Affiliations:** 1 Department of Psychology, Kroc Institute for International Peace Studies, University of Notre Dame, 390 Corbett Family Hall, Notre Dame, IN 46556, USA; mcgargano@nd.edu; 2 Department of Psychology, University of Zagreb, Ivana Lucica 3, 10000 Zagreb, Croatia; dean.ajdukovic@ffzg.hr; 3 Department of Psychology, Faculty of Philosophy, University of Belgrade, 11000 Belgrade, Serbia; 4 Psychosocial Innovation Network, 11000 Belgrade, Serbia

**Keywords:** migration, mental health promotion, refugees, transit, MHPSS, Delphi method

## Abstract

Most interventions for mental health and psychosocial support (MHPSS) have been developed in contexts and with populations that differ significantly from the realities of migration. There is an urgent need for MHPSS in transit; however, transit-specific aspects of MHPSS provision are often neglected due to the inherent challenges transit poses to traditional conceptualizations of practice. The Delphi method, which consisted of three iterative rounds of surveys, was applied with the goal of identifying challenges to and adaptations of MHPSS in the transit context. Twenty-six MHPSS providers working with refugees in 10 European transit countries participated; 69% of participants completed all three survey rounds. There was consensus that a flexible model of MHPSS, which can balance low intensity interventions and specialized care, is needed. Agreement was high for practice-related and sociopolitical factors impacting MHPSS in transit; however, the mandate of MHPSS providers working in the transit context achieved the lowest consensus and is yet to be defined. There is a need to rethink MHPSS in the refugee transit context. Providing MHPSS to refugees on the move has specificities, most of which are related to the instability and uncertainty of the context. Future directions for improving mental health protection for refugees, asylum seekers, and migrants in transit are highlighted.

## 1. Introduction

According to UNHCR reports, by the end of 2020, the number of people who were forced to leave their home countries in order to escape war, persecution, and human rights violations and to search for safety reached 82.4 million [1]. The current refugee crisis in Europe has lasted for years now, and recent events indicate that new challenges are to be expected [2]. As noted at the 20th Berlin Conference on Refugee Rights in 2020 [3], this crisis has long been exacerbated by inadequate investment in sustainable asylum systems. As a result, states increasingly seek to avoid their human rights obligations to asylum seekers and to place human rights at odds with border management. This rhetoric and the policy choices stemming from it have increased the time spent in transit, leading to substantial consequences for the daily lives and wellbeing of refugees, migrants, and asylum seekers.

It is important to note that, while crisis language is often used to frame refugee movements, for refugees, each decision to move is part of a much longer journey [4]. After leaving their home countries, refugees, asylum seekers, and migrants (For better readability and simplicity, the term refugee will be used throughout the text regardless of individuals’ legal status) often try to reach destination countries in Western Europe, where they seek international protection and can start rebuilding their lives. One of the main land transit routes refugees are using, with over 150,000 crossings in 2019 [5], is the Western Balkan route, which includes Turkey, Greece, Bulgaria, North Macedonia, Montenegro, Albania, Croatia, Bosnia and Herzegovina, and Kosovo (This designation is without prejudice to positions on status and is in line with UNSCR 1244/1999 and the ICJ Opinion on the Kosovo declaration of independence [5]. After EU migration governance policies sought to close the Balkan route following the spring of 2016, refugees are increasingly pushed to try “The Game”, informal travel into Western Europe [6]. For the majority, transit from their homes to the destination countries does not represent a journey but rather the advent of transit as a new reality of living on the move. It has been documented that transit can last from several months to several years [7]. Transit usually includes a long and risky route across the sea and land where refugees are exposed to detention or extended stays in camps [8] and illegal pushbacks [9], as well as physical and psychological violence [7,10,11], human trafficking [12], and other life-threatening situations [13]. In addition, prolonged periods of time in transit usually entail poor housing, limited access to essential services such as healthcare [14,15], discrimination by the local community, and separation from family and friends, which can lead to a lack of social support, isolation, and loneliness [13,16]. The urgency of improving conditions in transit is evidenced by the conclusions of the Second Berlin Conference on Libya in 2021, which called for increased respect for human rights, addressing human trafficking and the use of detention, and the development of a comprehensive approach to migration in the Libyan transit context [17].

The situation of protracted transit, in addition to bringing numerous risks for refugees, also leads to various challenges for countries along transit routes who are obliged to provide protection and care to people currently residing in their territories. These responsibilities include ensuring protection of rights, access to adequate shelter, food, and other basic living necessities, and access to healthcare and social services, as well as to the asylum procedure for those seeking international protection. The provision of these protections and services is even more difficult for countries that are already struggling with limited resources, particularly when it comes to health and social protection systems, which is often the case in countries along transit routes. For many state systems, the current refugee crisis has highlighted preexisting gaps in social services and infrastructure as well as the need to rethink existing practices and policies. This is not only due to limited resources, but also stems from specific characteristics that are inherent in the transit context and the population of concern, which may call into question if and how some traditionally conceptualized services respond to the needs of refugees on the move.

### 1.1. Implications of the Transit Context for Existing Mental Health and Psychosocial Support Practices and Policies

Most interventions for mental health and psychosocial support (MHPSS) have been developed and tested in contexts that differ significantly from the realities of refugee life on the move. Namely, the unpredictability of circumstances in the transit context presents challenges for informed treatment planning and, accordingly, deciding which interventions should be applied. In addition, health care systems in most transit countries are often under-resourced and provide limited access to MHPSS services, while services funded by international agencies and civil society usually lack continuity and sustainability. Therefore, the standard frequency and duration of treatment may not be achievable. Typically, there is a shortage of trained interpreters and cultural mediators, as well as MHPSS practitioners sensitized for work in the context of migration. Furthermore, refugees’ short duration of stay in one country and the lack of cross-country cooperation mean that it is not possible to ensure continuity of care. All of these factors pose challenges to the way MHPSS practice has traditionally been conceptualized in the stable and well-resourced contexts of European and North American destination countries.

Additional challenges to traditional MHPSS practice stem from other characteristics of the transit context and the population in question, including the high prevalence of mental health difficulties that have been identified in refugee populations [18,19,20,21,22] due to continuous exposure to stressful and traumatic experiences [7,23,24,25,26], the role of interpreters and cultural mediators in providing care [27,28], ethical dilemmas in conducting research [29], and a deficit of cultural understanding in MHPSS treatments [30]. Finally, it is important to investigate to what extent existential threats, including numerous legal and socio-economic issues refugees are facing [16], may limit the overall effectiveness of MHPSS interventions [31]. Under these circumstances, the role of MHPSS practitioners can hardly be limited to traditional norms when defining roles and boundaries. It can be assumed that MHPSS practitioners in the transit context are more involved in advocacy work, as well as cooperation with multidisciplinary teams that are jointly providing protection and support to refugees.

### 1.2. The Present Study

The aim of the current study was to identify challenges and areas requiring adaptation within MHPSS in the refugee transit context. Additionally, the study aimed to provide essential evidence to inform the adaptation of MHPSS guidance to the transit context in order to improve future research and practice.

## 2. Materials and Methods

### 2.1. Delphi Process

The Delphi method [32] is commonly used to harness the value of experts’ opinions and experiences in order to identify areas of consensus or lack thereof, which can inform future research, practice, and decision-making. It is particularly useful in situations where there is insufficient evidence or in new areas of research. In the field of psychology, the Delphi method has been used to create guidelines for post-disaster psychosocial care [33], to develop training programs [34], and to identify research priorities [35]. The present study adopts this method with the goal of using experts’ opinions to identify obstacles and therefore areas that need to be addressed in future research and practice in the provision of evidence-based MHPSS services to refugees.

### 2.2. Statement Development

For the purpose of the present study, a group of experts convened to develop the initial statements. This group consisted of members of the Research Working Group of the Consortium on Refugees’ and Migrants’ Mental Health (CoReMH). The Consortium on Refugees’ and Migrants’ Mental Health was established in 2020 with the goal of facilitating international cooperation between mental health experts and practitioners working on the ground with refugees in the transit context. The CoReMH is devoted to identifying and addressing prominent issues in mental health protection for refugees, asylum seekers, and migrants, through evidence-based practice, research, and advocacy work. The authors are members of the CoReMH. In total, eleven experts from Serbia, Croatia, Turkey, Italy, Bulgaria, and Kosovo, 91% of whom were female, participated in the statement development process. Initially, open-ended questions and general topics were developed during the first online meeting. Subsequently, the initial set of statements was collaboratively produced, and the subsections of the survey as well as methodological details such as the inclusion criteria and sampling method were discussed and defined. The resulting initial survey draft was sent to all experts who participated in statement development as a Google form. They were asked to provide their opinion on the inclusion and wording of each statement by choosing between “include as is”, “remove completely”, or “reword/change”. If they chose the third option, they were asked to include their reasoning or suggested changes. Further, space for additional comments, questions, ideas, or concerns was provided at the end of each section, as well as at the end of the survey to allow the experts to express their opinions and provide suggestions. The resulting data were used to inform a new draft of the survey and a second experts’ meeting was convened. At this meeting, the final version of the Initial Survey was completed.

Ultimately, the Initial Survey consisted of 12 sections. At the beginning of the survey, key terms were defined, and demographic questions were asked. Section 1 of the Initial Survey focused on understanding the mandate of mental health practitioners in the transit context and how they define their job. Sections 2–4 asked participants to consider the impact of the transit context on MHPSS interventions, identify contextual challenges to MHPSS, and consider what does and does not work in this context. Section 5 explored how the professional roles, settings, and boundaries in transit are similar to or different from those in a standard context. Sections 6–8 delved into evidence-based practice in the transit context, as well as ethical and methodological challenges that are inherent when working with refugees, and realistic measures of assessing effectiveness. Sections 9–11 focused on the mental health of practitioners themselves; specifically, they examined the impact this work has on them, the way that they perceive the value and efficacy of their interventions, and their cooperation with other providers. Section 12 consisted of two open-ended general conclusion questions regarding what else people should know about MHPSS provision in the transit context and why rethinking MHPSS of that context may be plausible.

The surveys were administered using Google Forms.

### 2.3. Participants

The inclusion criterion for participants was defined as having a minimum of one year of experience working in the provision of MHPSS services to refugees in the transit context. Participants were recruited through the CoReMH, as well as other providers in members’ networks, using a snowballing sampling strategy. During Round 1, 26 participants completed the survey of which 3 self-identified as male (11.5%) and 23 identified as female (88.5%). Participants’ ages ranged from 24 to 70 years (mean = 36.5, median = 32.5, sd = 12.7) and they were from 10 countries (Albania, Bosnia and Herzegovina, Bulgaria, Croatia, Greece, Hungary, Italy, Kosovo, Serbia, and Turkey). Years of experience ranged from 1 to 29 (mean = 6.8, median = 4.5, sd = 7.9). The vast majority of the participants (*n* = 22) described their professional and educational background as related to psychology, including clinical psychology, psychological rehabilitation, psychological counseling, psychiatry, psychotherapy, and having a master’s degree in psychology. Other professions included workshop management, social work, child protection, academia, and nonprofit leadership. During Round 2, 24 of the original participants completed the survey with an acceptable attrition rate of 7.69%. Eighteen of the original participants completed the Round 3 (Final) Survey with an overall attrition rate of 30.77% from Round 1.

### 2.4. Procedure

The study included three rounds of data collection. During the first round, the participants were asked to rate their level of agreement with each statement using a 10-point Likert scale where 1 indicated strong disagreement and 10 indicated strong agreement. In addition, they were asked to explain their rationale, leave comments, or suggest the addition or removal of statements. The statements that reached high consensus after the first round were not included in the following survey rounds, meaning that the number of statements being assessed decreased in each round. The statements that had not reached sufficient consensus were reviewed and adapted to be clearer and to better capture the concept in question or deleted, based on statistics and qualitative data obtained in the first round.

Panelists were then asked to complete Round 2 of the survey and assess 87 statements. This time they were provided with contextual data: each item was accompanied by the average agreement score and the level of consensus from Round 1. Each participant was also able to see the score they had assigned to items in the previous round. They could choose to keep their previous score or change it based on the contextual information provided and were asked to justify their reasoning for this decision. The same process was repeated in Round 3 when 30 statements were assessed with a final survey.

### 2.5. Data Analysis

Agreement levels for each statement were based on the mean of the assessment the panelists chose on the 10-point scale. This agreement level is a measure of the importance attributed to each particular statement related to MHPSS in the transit context.

The consensus level reached for each statement was derived from the coefficient of variation (CV), a measure that takes into account both mean and standard deviation [36]. The CV is inversely related to the level of consensus such that a smaller CV indicates less variation in individual scores for a given statement and is evidence of a higher level of consensus. Statements were considered high or low consensus if they had a CV of less than 0.2 or above 0.2, respectively. Only statements that fell into the high consensus category (CV < 0.2) were retained in the final list of statements that reached consensus (Appendix A (Table A1).

RStudio Cloud (Version 4.0.3, RStudio Team, Boston, MA, USA) [37,38,39,40,41] and Google Sheets (Google LLC, Mountain View, CA, USA), were used for all statistical and data visualization purposes.

## 3. Results

### 3.1. Round 1

The first round of the Delphi study indicated a fairly high overall level of agreement with the contents of the initial 92 statements: the average rating across all participants and statements was 7.71 points on the 10-point scale. A high level of consensus was achieved for 27 statements.

Out of the remaining statements, based on statistics and suggestions from the participants, 34 were reformulated, 16 were deleted, and 13 were kept in their original form. Based on the qualitative data collected in the survey, one new section (Section 13) consisting of 28 statements was introduced, entitled “Impact of Sociopolitical Context on MHPSS in the Transit Context,” which focused on policies and other structural characteristics of the transit context.

### 3.2. Round 2

As in the first round, there was a high overall level of agreement with the contents of the statements included in the second-round survey. Of the 87 statements panelists were asked to rate, 46 met requirements for high consensus.

Of the remaining statements, 5 were reformulated, 12 were dropped, and 26 were kept in their original form.

### 3.3. Round 3

In Round 3, high consensus was reached for 20 statements. Ten statements failed to achieve a high level of consensus.

### 3.4. Emerging Themes

In total, 93 statements reached consensus. These statements are listed by theme in Appendix A (Table A1) To the right of each statement, the average score is provided as a metric of how strongly participants agreed with the statements that reached consensus.

The highest overall average score was observed for the importance for close cooperation between MHPSS practitioners from different countries on the route (M = 9.39). Of all the statements that reached consensus, only two had average scores below 7, indicating an attribution of lower importance to these particular aspects of MHPSS. The first focused on whether the ethical, practical, and methodological concerns limit possibilities for conducting research in transit (M = 5.89); the second addressed whether the transit context decreases clients’ motivation to engage with MHPSS services (M = 6.61).

Three main themes emerged from our findings. First, the failure to reach consensus on certain statements related to the mandate of MHPSS providers working in the transit context revealed that this professional role and identity remain unclear and require further definition. For example, when considering which types of interventions make sense in the transit context, panelists showed the highest support for occupational workshops and activities being an important part of MHPSS for people on the move (M = 9.31) but failed to agree upon two typically central tenets of MHPSS providers’ jobs: providing specialized services for diagnosed mental health disorders (CV = 0.34) and helping people process potentially ongoing traumatic and stressful experiences (CV = 0.30). Despite the lack of consensus on these two statements, study results show that there is considerable agreement on certain aspects of providers’ mandate. The most strongly agreed upon aspects were raising awareness of the importance of MHPSS for people on the move (M = 9.00), recognizing and validating clients’ existing capacities and strengths (M = 9.13), and connecting clients with other resources (M = 9.19). These high-agreement statements can provide insights into how practitioners think about their role in transit and can serve as a starting point for future discussions on the mandate of MHPSS providers in transit that seek to explore and address the identified areas of disagreement.

Second, a trend in the data across several sections was that many features inherent in the transit context present challenges to the ways in which both practice and research are usually carried out. Of the statements in Section 2, which focused on the impact of transit on MHPSS interventions, the importance of a flexible model of care that could meet clients’ needs wherever they are reached the highest level of agreement (M = 8.83). When considering challenges to MHPSS in transit, participants most strongly agreed that MHPSS interventions should be adapted to consider the impact of circumstantial factors in transit (M = 8.96). This complicates and presents both ethical and practical challenges for the application of methods for treatment and data collection in transit that were originally developed for use in contexts with different resources and were likely tested on populations with different needs and backgrounds. Nevertheless, there was a high level of agreement regarding the need for more evidence on the effectiveness of MHPSS intervention implementation in transit (M = 8.42), which highlights that, despite the numerous challenges identified, providers agree that more transit-specific research, which could enable evidence-based practice, is urgently needed.

Third, a high level of agreement was exhibited for most statements from Section 13 on the impact of the sociopolitical context on MHPSS in transit. Specifically, border-related violence (M = 8.78), the high level of uncertainty (M = 8.70), the undesired extension of time spent in transit (M = 8.70), stress surrounding the asylum procedure and the low likelihood of obtaining asylum (M = 8.39), and the present political context (M = 8.22) were all identified by providers as stressors, which negatively impact the mental health of people on the move. These findings highlight the importance of considering social determinants of mental health, including environmental conditions created or reinforced by migration governance policies, in the design and provision of MHPSS services in transit.

## 4. Discussion

The main aim of the current study was to identify challenges to and areas for adaptation within MHPSS services in the refugee transit context as well as to provide the evidence needed to guide that adaptation. The study findings fall into three broad themes.

### 4.1. Identity of MHPSS Providers

This study showed there is a common understanding of the importance of some aspects of MHPSS providers’ mandate. Namely, the panelists overwhelmingly assessed as important and agreed that their mandate involves maintaining clients’ mental health and wellbeing, helping people survive and cope in healthy ways, raising awareness of the importance of MHPSS, and bolstering extant resilience capacities. Interestingly, the highest level of agreement in the mandate section corresponded to connecting people on the move to other resources, highlighting the multiplicity of roles that MHPSS providers are expected to play in the transit context.

Surprisingly, the experts did not agree that the job of MHPSS practitioners working with refugees in transit should include treating diagnosed mental health disorders. Upon reviewing the qualitative data linked to this topic, it was found that those who agreed with the statement mainly argued that this was a central part of their work, as it is essential to prevent deterioration of mental health, especially for those presenting with severe symptoms (e.g., “It is a necessary part of our job. Of course it will be done in a team (e.g., in collaboration with the psychiatrists), but if this is not the job of MHPSS practitioners providing MHPSS support to people on the move, I do not understand whose it should be”). Conversely, those who did not agree with the statement expressed concern about limited resources, potentially inadequate training and competencies, ethical issues in medicalizing symptoms, and the lack of continuity of care. It is important to highlight that these factors have also been identified as legitimate concerns by past research, which has found harmful effects linked to these factors for both providers and clients. Low resources are often linked to increased caseloads for providers, which has been shown to be associated with emotional exhaustion and burnout in community mental health care providers [42]. Inadequate cultural competency training and treatment adaptation has been shown to cause harm, and the dominance of the biomedical model of mental health has been challenged, especially in work with refugee populations [43].

In addition, there was a lack of consensus around the importance of helping refugees to process potentially ongoing trauma. As with the previous statement, those who agreed expressed that this was a large part of their job and that it was their responsibility to provide such support (“This is probably the primary job and it’s the job that takes up the most time in this kind of setting.”). Those who were opposed argued that it is more appropriate to help the refugees process trauma when people have settled in a destination country and achieved a basic state of safety and stability (“Besides the basic interventions, it is best to start treatment once the person reaches a destination country and other preconditions for continuous therapeutic work and life circumstances are stabilized”). Whether providers considered this an important aspect of their mandate or not in the transit context, they emphasized the need to acknowledge individual preferences across clients and the beneficiary’s perspective (“Processing of traumatic experiences in my opinion should not be instigated by the MHPSS practitioner if it is not on the agenda of the beneficiary.” “If they want to, there are many clients who do not wish to go through trauma with MHPSS professionals, and that has to be respected”). The unanticipated contentiousness of these statements demonstrates how, in the transit context, even the most basic functions of mental health care provision are called into question.

There are several potential causes for these findings. It is possible that the lack of universal standards on required qualifications for MHPSS care providers poses a concern regarding the expertise needed to ensure the required quality of care for refugees who have experienced several traumatic events. This could explain why some providers are hesitant when it comes to the more sensitive aspects of MHPSS work, such as providing treatment to persons with mental health disorders or processing traumatic experiences. In addition, it seems there are dilemmas among care providers about whether certain types of MHPSS interventions, primarily specialized care, should be applied before refugees arrive at their destination and achieve some form of life stability. However, the importance of providing an opportunity to process ongoing trauma whenever it is introduced by the client was highlighted (“It is not ours to decide when the need for support in processing traumatic and stressful experiences will appear, but to be ready to provide support whenever that happens. And experience says it might happen while people are still in transit”). Interestingly, there is agreement, both within and outside of our study, on the provision of mental health services in the post-migration context. Healthcare professionals have stressed the importance of mental health care provision for newly arriving refugees as well as strengthening continuity of care [44]. A qualitative study conducted with refugees who had recently arrived in seven European countries also identified psychological distress as a main health problem, as well as a need to address the deficit in continuity of care for this population [45]. Both provision and continuity of mental health care are equally important in transit, yet they may be even more challenging to achieve in this context due to time and resource limitations.

The short time refugees tend to spend in transit countries could also account for the avoidance of the aforementioned interventions. However, previous studies have shown that time spent in transit varies from several months to several years [7], so it is hard to justify the sustained lack of specialized care observed in this context. Finally, a potential explanation may be the recent paradigm shift from more substantial specialized treatment to low intensity peer-support models of MHPSS [46], which may have impacted the way care providers’ understand the expectations and goals of their work.

Furthermore, the methods that are currently promoted by most international bodies and donors supporting MHPSS services, particularly in low- and middle-income countries, mainly include basic psychosocial support. Individual specialized care is rarely funded by international donors and is consequently rarely part of project-based MHPSS care providers’ mandates. This, in the long run, may influence the way MHPSS care providers conceptualize their professional identity and, accordingly, the way they orient their practice towards or away from treating mental health disorders and trauma (“I think that while trying not to stigmatize people with mental health difficulties we slip into the opposite extreme and have started stigmatizing mental health disorders as such. They might be disorders, but they can also be treatable, and people should be encouraged to address them, and MHPSS care providers should provide support along this process”). Overall, this study showed polarization among care providers, which highlights the urgent importance of rethinking core aspects of MHPSS care providers’ identity and their understanding of their mandate.

When it comes to other aspects of MHPSS practitioners’ mandate, including setting, roles, and boundaries, there was agreement that the transit context differs from that of standard work in several ways. These include lack of proper premises for delivering MHPSS services, working with interpreters, and MHPSS practitioners balancing multiple roles in relation to their clients, all of which make maintaining boundaries more difficult than in the standard context. Furthermore, this study revealed that advocacy work is conceptualized as one of the core aspects of MHPSS work in the transit context. This includes working in multidisciplinary teams, advocating for clients’ rights, informing policy development, and striving to achieve a political impact that will be protective for the mental health of refugees. Thus, MHPSS providers’ responsibility for education on human rights violations and for supporting clients in knowing their rights and reporting any violations in order to avoid perpetuating an inhumane system was highlighted (“These systemic stressors are exactly the reason why MHPSS providers should engage with and actively contribute to organized advocacy efforts and politically engaged consultancy, campaigning, publishing, etc., which is needed for an influential impact on political decision making”). Therefore, the study revealed an evolving aspect of MHPSS providers’ mandate, which emerged as a response to job requirements in such precarious and dynamic circumstances and confirmed the need to rethink and adjust definitions of the role of MHPSS providers in the transit context.

### 4.2. Specifics of MHPSS in the Transit Context

The study revealed a need to define suitable MHPSS interventions for the transit context. There was support for developing a more flexible model of care ranging from community-based to specialized care, in accordance with past research on meeting mental health needs in the transit context [47]. This model should be adjusted to the needs of people on the move, which could be enabled by continuously assessing needs and by including refugees themselves in the design and provision of MHPSS services in the transit context. Furthermore, this model should be adjusted to transit-specific systemic factors, such as unpredictability, lack of control over time, and major life events that refugees might experience during transit, all of which are related to mental health and, consequently, impact the types of MHPSS interventions that should be applied in the transit context. These findings align with those of past research, which has underscored the importance of incorporating an understanding of environmental factors into MHPSS service provision [48]. The transit-specific contextual factors our study identified could add to the cumulative load of environmental stress a person of refugee background experiences as posited by Kashyap and colleagues’ (2021) “Psychological Interaction with Environment (PIE) Matrix Model” of refugee mental health and should be investigated further in the future [48]. Additionally, several other material factors limiting the efficacy of practice in the transit context, such as lack of funding and referrals for people in remote locations where support is not available, were considered important. This echoes findings from other researchers who have emphasized the need to increase accessibility to care in transit [47].

It was also pointed out that trust, which is foundational for successful MHPSS interventions, is much more difficult to establish in the transit context given the uncertainty created by sociopolitical factors. Our qualitative data offers an example of how some providers conceptualize the impact of transit-related factors on the establishment of trust (“Re-traumatization, exacerbation of symptoms, and impaired functionality impacted trust in MHPSS experts and services. [There could be] serious deterioration of mental health due to interventions which shouldn’t be implemented before basic preconditions (such as safety/stability/basic needs) are met”).

Other researchers have highlighted the importance of strengths, values, and action-based intervention approaches in the transit context [49], an approach that was also supported by our qualitative data (“In my opinion [processing traumatic and stressful experiences] should mean focusing on survival, successful coping, strengths and resources”). The variance in ideas about which types of interventions are and are not appropriate in transit reflects an ongoing debate in the field regarding how best to adapt MHPSS care provision to the specific contextual factors of life on the move.

These contextual factors were also seen as impacting care providers’ mental health and creating feelings of hopelessness, guilt, and anger (“It was often the case to have questions on migration policies within psychological sessions. It caused and triggered my feelings of shame and helplessness, and the awareness that I am also part of that system which failed to provide protection and abandoned people, as well as the fact that I did not do enough to change it.”).

Furthermore, the need to conduct more research within the transit context to inform practice as well as for additional training for MHPSS practitioners on research methodology, in order to support future evidence-based practice in transit, was recognized. Moreover, the panelists agreed that there is a need to define what can be considered “evidence” when discussing evidence-based practice in the transit context.

However, despite the numerous contextual limitations, there was a high level of agreement on the perceived value and effectiveness of MHPSS in the transit context, which can be attributed to a belief that MHPSS in the transit context can make a difference, regardless of the myriad obstacles inherent in transit.

### 4.3. Sociopolitical Factors

Another insight from this study is the importance of considering the impact of the sociopolitical context on MHPSS in the transit context. Sociopolitical context and European migration and border governance policies were recognized as risk factors that impact refugees’ mental health and the effectiveness of MHPSS interventions. Moreover, numerous factors introduced by the sociopolitical context represent under-researched areas and are typically not accounted for in MHPSS. These include, but are not limited to, border-related violence, pushbacks, the undesired extension of time spent in transit, high level of uncertainty, lack of legal and safe pathways for refugees, unpredictability and unfairness of migration procedures, police violence and exposure to violence from criminal groups, and stress surrounding the asylum procedure, including potential retraumatization [50] and the low likelihood of obtaining asylum. These findings largely align with past work on social determinants of health, which has demonstrated that the environmental conditions in which people live, shaped by systemic inequities and structural violence, can impact health between and within societies [51]. The concept of social determinants has also been applied to mental health, highlighting the relationship between social inequalities and exposure to risk factors for many mental health disorders [52]. Qualitative data from many participants in our study highlight providers’ agreement regarding how foundational social determinants, including sociopolitical factors, are in both shaping refugees’ daily lived experiences and MHPSS care provision. (“For any disadvantaged population, socio-economic and political factors that lead to and maintain discrimination and racism play a role in mental health and wellbeing, particularly if disadvantages and inequalities are trans-generational”).

This study also highlighted that the stressful and traumatic experiences refugees go through during transit constitute another round of trauma, sometimes consisting of more traumatic events than were experienced in the country of origin, whose negative effects on refugees’ mental health have been demonstrated in previous studies [7]. In addition, the study provided insights into how the wider sociopolitical context is represented in refugees’ narratives and shared during psychological support sessions as well as how this can consequently trigger additional stress for refugees (“I also would like to emphasize that potential retraumatization, low likelihood of obtaining asylum, bureaucracy of seeking asylum, limited access to basic needs, health, education and high level of uncertainty, inconsistent policies have a greater effect on the mental health of this vulnerable group”). While, to our knowledge, social determinants of mental health specific to transit have not been studied, researchers have investigated social determinants of mental health in the post-migration and resettlement contexts. Chen and colleagues found evidence to suggest that the social environment in the post-migration context is essential for humanitarian migrants’ mental health [53]. Others have concluded that interventions should incorporate elements designed to address broader social, political, economic, and material conditions of post-migration life, a conclusion also reached in our study [54]. Similarly, Li, Liddell, and Nickerson discussed how sociopolitical factors can impact refugees’ psychosocial functioning in the post-migration context [55]. Unfortunately, there is evidence that, as a result of the COVID-19 pandemic, the existing inequalities driving social determinants of mental health for refugees, as well as challenges to accessing MHPSS services, have been exacerbated in the post-migration context [56].

Our study findings on the asylum process as a stressor are supported by past research, which has both found that the asylum procedure is a source of stress and recognized policies related to migration as having shaped many social determinants of health for migrant populations [54,55,57]. Participants in our study repeatedly focused on the potential impacts of the asylum process and related stressors on refugees’ wellbeing. This is evidenced by both survey data, for instance the high agreement with statement 81 in Appendix A (Table A1), as well as qualitative data (“While waiting for asylum approval and going through interviews and new procedures, people often experience stress that affects their memory, sleep and relationships with people”). While several social determinants overlap between transit and post-migration contexts, there is a need for further research that could identify and evaluate ways to address transit-specific social determinants of mental health, such as border-related violence.

The study points out that mental health protection for refugees on the move cannot be ensured by interventions at the individual level alone, without addressing wider systemic and sociopolitical factors, as it would be hard to speak about the long-term effectiveness of MHPSS interventions (“[The] global political situation, asylum policies and rules, and closing borders often cause additional distress and disrupt wellbeing and progress that has been made”).

### 4.4. Study Limitations and Future Directions

The Delphi method included 26 panelists, of which 18 assessed the statements in all three rounds. The retention rate of 69.23% of the initial pool of panelists throughout the three iterations and over a two-month data collection period probably reflects more dedicated and perhaps more experienced providers. The uneven representation of female and male panelists, although a possible source of bias, in fact broadly corresponds to the situation in the MHPSS field in countries on the refugee route to Western European destination countries, where most often the providers are female. The panelists had a variety of professional backgrounds and many of them had an activist history. This may have impacted the lack of consensus on statements regarding specialized care provision, given that some panelists did not have competencies in this area. The uneven number of statements across sections of the Delphi survey reflects the possible bias of the group of experts who identified the initial areas of MHPSS provision in the transit context to be assessed and their concern with the vague definition of the mandate and job of providers under these circumstances. However, during the three Delphi iterations, the panelists had the opportunity to influence the formulation of statements, their rejection, and even the inclusion of new sections.

## 5. Conclusions

Ultimately, this study reinforces the importance of recognizing how the transit context impacts the standard view of MHPSS that is typically provided in much more stable and well-resourced conditions.

The study highlights several important aspects of the current state of the field that need to be reconsidered. First, there is a need for a clearer understanding of the versatile and multidimensional mandate of MHPSS practitioners in the refugee transit context. Second, the importance of considering specificities of the transit context when designing and implementing MHPSS services is highlighted. To this end, providers advocated for a more flexible model of MHPSS that can adapt to the changing needs of refugees on the move and meet them where they are at. This model should be neither so ‘zoomed-in’ on specialized care that it loses sight of the vast number of people needing other kinds of support, nor so ‘zoomed-out’ that it loses sight of the individual needs of each person within the range of MHPSS possibilities. Third, the study reveals the importance of addressing both individual and systemic risk factors for mental health in order to ensure sustainable and comprehensive mental health protection for people on the move. Finally, the study pointed to several key future directions for improving MHPSS in the transit context. These include a need for closer cooperation between MHPSS practitioners from different countries along the route to enable continuity of care, as well as to exchange knowledge, experiences, and lessons learned. The need for collaboration is also evidenced by calls for the production of guiding documents, which could inform research, practice, and advocacy work in the transit context, thus enabling appropriate mental health protection and care for refugees on the move.

## Data Availability

The data and R code for the study are available upon request from the corresponding author.

## Appendix A

**Table A1:** Statements that reached consensus and average score for each of the 93 statements.

Section 1: Mandate of MHPSS Practitioners in the Transit Context	Average
1. The job/mandate of MHPSS practitioners working with people on the move in the transit context is to connect people with other resources (legal, medical, practical, etc.).	9.19
2. The job/mandate of MHPSS practitioners working with people on the move in the transit context is to recognize and validate internal capacities, strengths, and successes.	9.13
3. The job/mandate of MHPSS practitioners working with people on the move in the transit context is to raise awareness of the importance of MHPSS for people on the move.	9.00
4. The job/mandate of MHPSS practitioners working with people on the move in the transit context is to encourage positive coping mechanisms.	8.89
5. The job/mandate of MHPSS practitioners working with people on the move in the transit context is to maintain/stabilize the mental health and well-being of the client.	8.89
6. The job/mandate of MHPSS practitioners working with people on the move in the transit context is to provide a space of safety and interpersonal trust.	8.83
7. The job/mandate of MHPSS practitioners working with people on the move in the transit context is to help people on the move to cope with their psychological trauma.	8.78
8. The job/mandate of MHPSS practitioners working with people on the move in the transit context is to help people develop a toolbox for their well-being that they can take with them on the route.	8.69
9. The job/mandate of MHPSS practitioners working with people on the move in the transit context is to prevent deterioration of the client’s psychological condition.	8.65
10. The job/mandate of MHPSS practitioners working with people on the move in the transit context is to help the client survive and make sense of the situation.	8.54
11. The job/mandate of MHPSS practitioners working with people on the move in the transit context is to triage in order to stretch the available resources to address the most urgent needs.	8.31
12. The job/mandate of MHPSS practitioners working with people on the move in the transit context is to mitigate destructive coping mechanisms such as substance abuse.	8.28
13. The job/mandate of MHPSS practitioners working with people on the move in the transit context is to strive for improvement of their psychological condition.	8.22
14. The job of MHPSS practitioners working within the transit context differs from standard work.	8.15
15. The job/mandate of MHPSS practitioners working with people on the move in the transit context is to screen for mental health disorders and refer clients to the appropriate specialized services.	8.13
16. Advocacy is an important part of the job/mandate of MHPSS providers working in the transit context.	7.87
**Section 2: Impact of the Transit Context on MHPSS Interventions**	
17. In the transit context, it is important to have a flexible model of care, including a range of specialized and peer/community-based services that can meet individual clients where they are at.	8.83
18. It is important to assess the risk of retraumatization to the people on the move providing translation, interpretation, and cultural mediation services with other people on the move.	8.74
19. Positive effects of MHPSS cannot be expected to be at the same level in situations where basic safety and survival needs have not yet been met, which is sometimes the case in the transit context.	8.65
20. Care should be provided even if the MHPSS interventions cannot be completed due to clients leaving the country.	8.61
21. It is important to assess the risk of retraumatization and other harm for people on the move providing and receiving peer support services.	8.57
22. Psychiatric care adjusted to the transit context is an important component of MHPSS in the transit context.	8.52
23. Expected results and overall reach of MHPSS interventions should be adjusted to the limitations and characteristics of the transit context.	8.48
24. There is a need to define suitable interventions for the transit context.	8.48
25. MHPSS should be adapted as the needs of people on the move change.	8.35
26. Peer based support is an important component of MHPSS in the transit context.	8.22
27. Even though there is likely a strong impact of circumstances, it is possible to successfully implement MHPSS interventions in the transit context.	8.17
28. MHPSS services in the transit context require unexpected adaptations.	7.96
29. Standard MHPSS interventions applied in the transit context cannot be as effective as they are in the standard context.	7.78
30. MHPSS interventions based on evidence from other populations and in other contexts can only be successfully applied in the transit context if they are properly adapted for that purpose.	7.78
31. There is a risk of doing harm by applying trauma-related interventions that cannot be completed due to the lack of available time in the transit context.	7.56
32. The transit context decreases clients’ motivation to engage in MHPSS services.	6.61
**Section 3: Challenges to MHPSS in the Transit Context**	
33. MHPSS interventions in the transit context should be adapted taking into consideration the impact of circumstances which may include major life events, stressors, and losses.	8.96
34. MHPSS interventions in the transit context should be adapted taking into consideration the impact of the lack of continuity of care.	8.77
35. Unpredictability of the transit context has an impact on the types of MHPSS which should be applied.	8.31
36. Unpredictability of the transit context has an impact on the overall effectiveness of MHPSS interventions.	8.04
**Section 4: What makes sense in the transit context?**	
37. Occupational workshops and activities (sports, arts, language classes) are an important component of MHPSS in the transit context.	9.31
38. Psychoeducational workshops and activities are an important component of MHPSS in the transit context.	8.81
39. Community based support is an important component of MHPSS in the transit context.	8.81
40. Psychotherapeutic interventions adjusted to the transit context are an important component of MHPSS in the transit context.	8.73
**Section 5: Setting, Roles, and Boundaries**	
41. When you are providing MHPSS in the transit context you are usually more than just a MHPSS practitioner to your client.	8.30
42. The setting in which interventions take place in the transit context differs from that of standard work.	7.83
43. The numerous systemic risk factors for mental health associated with transit, which are beyond the control of MHPSS practitioners (such as difficulty meeting basic needs and violation of social and economic rights), decrease MHPSS practitioners’ motivation for provision of MHPSS services.	7.65
44. Maintaining boundaries with clients is more difficult in the transit context than in standard work.	7.35
45. Limitations of the transit context and the numerous risk factors for mental health that it brings increase feelings of helplessness among MHPSS practitioners.	7.22
**Section 6: Evidence-Based Practice in the Transit Context**	
46. We need more evidence on the effectiveness of MHPSS services implemented in the transit context.	8.391
47. There is a need to define what can be considered “evidence” when discussing evidence-based practice in the transit context.	8.087
48. It is important to follow evidence-based treatment protocols as much as possible, taking into account the transit circumstances.	7.611
**Section 7: Ethical and Methodological Concerns of Research in Transit**	
49. It is important to include people on the move in the design and provision of MHPSS services in the transit context.	8.42
50. We should conduct research in the transit context despite the challenges it presents to standard research practices.	8.39
51. There is a need to conduct more research within the transit context in order to inform practice.	8.17
52. There is a need for additional training for MHPSS practitioners on research methodology and implementation in order to support future evidence-based practice in the transit context.	8.04
53. The typical methodological requirements from standard contexts are harder to meet in the transit context.	7.96
54. It is problematic to define treatment as usual (TAU) in the transit context due to lack of continuity of care, universal standards, and equal distribution of services on the route.	7.94
55. The ethical, practical, and methodological concerns of conducting research within the uncertainty of transit limit the possibilities for research in the transit context.	5.89
**Section 8: Realistic Measurements of Effectiveness**	
56. Well-recognized and commonly used measures and outcome variables should be included in assessing the effectiveness of MHPSS interventions in the transit context (e.g., well-being, symptoms of psychological difficulties, quality of life, etc.)	7.72
57. The level of evidence in evaluating the effectiveness of MHPSS interventions in the transit contexts should not be expected to be the same as in the standard context.	7.70
58. There is a need to introduce new, transit-informed outcome measures for assessing the effectiveness of MHPSS services in the transit context.	7.61
**Section 9: Impact on MHPSS on Practitioners**	
59. Awareness of the possibility that we are the first point where a person has the opportunity to receive care increases the motivation and responsibility for providing MHPSS services.	8.19
**Section 10: Perceived Value & Effectiveness of Interventions**	
60. MHPSS has the potential to be beneficial not only for mental health but also for overall quality of life of the people on the move.	8.885
61. MHPSS interventions are of particular importance in the transit context, due to numerous risk factors for mental health people in these circumstances are exposed to.	8.731
62. Experience of safety and responsiveness during MHPSS interventions can be of crucial importance for long-term wellbeing of people on the move.	8.692
63. Experience and insights from MHPSS may help people make informed and constructive decisions, which is of particular importance in the risky circumstances of the transit context.	8.462
64. Experience from MHPSS services/interventions can impact major life decisions of people in the transit context.	8.038
**Section 11: Cooperation with Other Actors and Experts**	
65. There is a need for closer cooperation between MHPSS practitioners from the different countries along the route in order to exchange knowledge, experience and lessons learned.	9.39
66. There is a need for closer cooperation between MHPSS practitioners from different countries along the route, in order to enable continuity of care.	8.92
67. The purpose and the importance of MHPSS in the transit context is adequately recognized by MHPSS professionals.	8.08
**Section 12: General Conclusions**	
68. The specific knowledge and experience of MHPSS practitioners working in the transit context should be consulted in migration policy development.	8.52
69. There is a need to integrate an understanding of how systemic factors can negatively impact the psychosocial wellbeing of people on the move, when conducting MHPSS interventions in the transit context.	8.30
70. There is a need for best practices and guiding principles for MHPSS provision in the transit context.	8.17
71. There is a need to rethink MHPSS in the transit context.	8.11
72. There is a need to rethink the role of MHPSS providers in the transit context.	7.83
**Section 13: Impact of the Sociopolitical Context on MHPSS in the Transit Context**	
73. MHPSS providers are not in a vacuum, they are part of local and international systems, and as such their jobs are interrelated with these systems.	8.78
74. Stress surrounding the asylum procedure (potential retraumatization, low likelihood of obtaining asylum, bureaucracy of seeking asylum) limits the effectiveness of MHPSS services provided to people on the move in transit.	8.78
75. Border-related violence (such as pushbacks) is a stressor, which can negatively impact the wellbeing and mental health of people on the move.	8.78
76. Unresolved existential threats (such as threats to basic safety) people on the move face have an impact on the effectiveness of MHPSS interventions.	8.72
77. MHPSS interventions in the transit context should be adapted taking into consideration the impact of unpredictability (especially of time/length of potential interaction of MHPSS providers with the clients).	8.72
78. The high level of uncertainty in the transit context is a stressor, which can negatively impact the wellbeing and mental health of people on the move.	8.70
79. The undesired extension of time spent in the transit context is a stressor, which can negatively impact the wellbeing and mental health of people on the move.	8.70
80. Migration policies that are informed by the experience of MHPSS experts in the transit context would be protective for the mental health and wellbeing of people on the move.	8.44
81. Stress surrounding the asylum procedure (potential retraumatization, low likelihood of obtaining asylum, bureaucracy of seeking asylum) is a stressor, which negatively impacts the wellbeing and mental health of people on the move.	8.39
82. In the transit context, existing inequalities and vulnerabilities are reinforced, potentially making certain groups more vulnerable for experiencing new traumatic events.	8.33
83. Evidence on the impact of migration-related stressors on mental health and wellbeing in the transit context should be used to shape migration policies in order to mitigate these stressors.	8.22
84. The present political context is a stressor, which can negatively impact the wellbeing and mental health of people on the move.	8.22
85. Unresolved existential threats (such as threats to basic safety) people on the move face have an impact on the types of MHPSS which should be applied.	8.13
86. Unpredictability of the transit context has an impact on the delivery of MHPSS interventions.	8.13
87. Major life events (leaving one’s country, separation from or death of family members), which often happen to the people in the transit context, have an impact on the types of MHPSS which should be applied.	8.04
88. When treating a person on the move, there is a concern related to what will happen and who will take over the provision of the MHPSS intervention after the person leaves the country.	8.00
89. European migration and border governance policies are a stressor, which can negatively impact the wellbeing and mental health of people on the move.	7.96
90. The impact of mental health on people on the move’s memory, decision-making, and ability to adapt to a new environment is not adequately taken into consideration in the creation of migration policies.	7.96
91. Major life events (leaving one’s country, separation from or death of family or friends), which often happen to the people in the transit context, have an impact on the effectiveness of MHPSS interventions.	7.94
92. The undesired extension of the time spent in the transit context limits the effectiveness of MHPSS services provided to people on the move in transit.	7.87
93. The high level of uncertainty limits the effectiveness of MHPSS services provided to people on the move in transit.	7.87
